# A post-marketing study to evaluate the safety and immunogenicity of a quadrivalent influenza split-virion vaccine in elderly people aged 60 years and older

**DOI:** 10.1186/s40794-024-00228-x

**Published:** 2024-09-15

**Authors:** Zengqiang Kou, Xiaoyu Li, Ti Liu, Bei Fan, Wenqi An, Wenjue An, Mingan Dang, Ke Zhang, Jingning Tang, Nan Zhu, Ruowen Pan

**Affiliations:** 1 Shandong Center for Disease Control and Prevention, Jinan, 250014 China; 2 National Institutes for Food and Drug Control, Beijing, 102600 China; 3 Hualan Biological Engineering Inc, Xinxiang, 453003 China; 4 Hualan Biological Bacterin Inc, No. 1-1, Hualan Avenue, Xinxiang City, Henan Province 453003 China; 5 Henan Center for Drug Evaluation and Inspection, Zhengzhou, 450008 China

**Keywords:** Quadrivalent influenza split-virion vaccine, Elderly people, Vaccination, Safety, Immunogenicity

## Abstract

**Background:**

Influenza remains a global public health concern. Understanding the vaccination-induced response in an aging population, which is susceptible and at high risk, is essential for disease prevention and control. Here, we report findings on the safety and immunogenicity of a quadrivalent influenza split-virion vaccine (15 µg/subtype/0.5 ml/dose) (hereinafter referred to as the “quadrivalent influenza vaccine”) in a population aged ≥ 60 years.

**Methods:**

This open-label, pragmatic post-marketing trial enrolled 1399 older adults to receive one dose of an approved commercially available quadrivalent influenza vaccine manufactured by Hualan Biological Bacterin Inc. (hereinafter referred to as “Hualan Bio”). Participants with contraindications for the vaccine were excluded, while poor health condition was acceptable. All vaccinated subjects experienced adverse events collection within 30 days and serious adverse events within 180 days post-vaccination. 25% subjects, selected randomly, underwent venous blood sampling pre-vaccination and 30 days after post-vaccination, for detecting antibody titers against each subtype of influenza virus by hemagglutination inhibition assay. The incidences of adverse events and antibody titers against each subtype of influenza virus were statistically analyzed using SAS 9.4.

**Results:**

No grade 3 adverse reactions occurred within 30 days post-vaccination. The incidences of overall adverse reactions, local adverse reactions and systemic adverse reactions were 3.79%, 2.86% and 1.00%, respectively. No serious adverse reactions occurred within 180 days post-vaccination. There were 350 subjects who completed venous blood sampling pre-vaccination, among whom 348 subjects completed venous blood sampling at 30 days post-vaccination for immunogenicity assessment. With respect to hemagglutination inhibition antibodies against influenza viruses H1N1, H3N2, BV and BY subtypes, at 30 days post-vaccination, the seroconversion rates were 87.64%, 75.57%, 73.28% and 78.74%, respectively; the seropositive rates were 93.97%, 98.56%, 79.31% and 95.40%, respectively; and the geometric mean increase (GMI) in post-immunization/pre-immunization antibodies was 24.80, 7.26, 10.39 and 7.39, respectively.

**Conclusion:**

One 15 µg/subtype dose of the vaccine had a good safety profile and elicited favorable immunogenicity among subjects aged ≥ 60 years. The results of this study indicate that Hualan Bio quadrivalent influenza vaccine strike balance between safety and immunogenicity, supporting unnecessity to increase dosage or inoculation frequency for further enhancing immunogenicity.

**Trial registration:**

Registered on ClinicalTrials.gov. Registration number: NCT06334510. Registered on 28/03/2024 (retrospectively registered).

## Introduction

Seasonal influenza is a globally common acute respiratory infection caused by influenza virus, which are usually classified as type A, B, C and D by their nucleoproteins and matrix proteins, and each type could be further divided into multiple subtypes. Typically, seasonal epidemics are caused by A/H1N1 and A/H3N2 and the B/Victoria and B/Yamagata lineages [1]. The prevention and control of disease remain a challenge due to the high antigenic variability of influenza virus and subsequent immune escape [2]. Rapid spread and the general susceptibility of influenza viruses also contribute to annual seasonal influenza epidemic, consequently result in substantial disease burden worldwide. Although the protection effectiveness of vaccine might be influenced by the antigenic mismatch to predominantly circulating strain, vaccine remains an important mean to bolster individual protection against influenza infection and for public health strategy. For those at greater risk of complications, such as pregnant women, children, the elderly and people with chronic medical conditions, WHO recommends annual vaccination [3]. Influenza incidence among elderly individuals (7.2%) is higher than that among adults (4.4%) [4]. Worse still for the elderly, influenza is more likely to lead to a high frequency of hospitalization [5]. From 2010 to 2012, the hospitalization rates of the elderly aged ≥ 65 years for acute respiratory infections were 89/100,000–141/100,000 [6]. Furthermore, the elderly are prone to face risk of serious complications, severe cases and death when suffering from influenza. The mortality rate is the highest among the elderly population compared to other age brackets [7–12]. A Meta analysis, involving 9 clinical trial and 62 observational studies for efficacy/effectiveness, estimated that the effect of influenza vaccine against influenza among elderly population aged 65 years or older was 58% (95%CI 34 − 73%) [13]. Another Meta analysis, involving 2,504,162 elderly persons from 95 trials, estimate the vaccine effectiveness (VE) against fatal or non-fatal complications of influenza was 28% (95%CI 26-30%), VE against typical influenza like illness (ILI) was 39% (95%CI 35-43%), and VE against laboratory-confirmed influenza was 49% (95%CI 33–62%) [14]. Results of previous study support the effectiveness of influenza vaccination to reduce risk of infection, complication and consequent clinical outcome among the elderly.

Up to now, trivalent and quadrivalent inactivated influenza vaccines have been approved for use in the elderly aged ≥ 60 years in China. Strength and immune procedure of all those products was: 15ug/dose for each subtype of virus, vaccinated with one dose (0.5 ml) prior to or during the influenza season. Compared with trivalent inactivated vaccines, quadrivalent products could offer broader protection, given the virological situation of cocirculating B strains. It is widely believed that vaccination-acquired immunity in elderly individuals might be relatively weak due to the decreasing count and proliferation capability of T lymphocytes, as well as a waning immune system with aging [15]. This could be reflected by the criteria issued by the Center for Biologics Evaluation and Research (CBER) of the Food and Drug Administration (FDA) and that by the European Medicines Evaluation Agency (EMEA) [16, 17], that is, influenza vaccination in adult populations should fulfil: (1) seroconversion rate > 40%; (2) seroprotection rate > 70%. That used in the elderly population should fulfil: (1) seroconversion rate > 30%; (2) seroprotection rate > 60%. In order to overcome the negative effect of a waning immune system on the vaccination-acquired immune response in the elderly population, the FDA-approved influenza vaccine enhances vaccine protection in the elderly by increasing the dosage and/or adding adjuvants [18, 19], such as inactivated vaccines with higher dosage of antigen (60.0 µg of hemagglutinin per strain), adjuvanted vaccines, and recombinant vaccines (45.0 µg of hemagglutinin per strain) [20].

The Hualan Bio quadrivalent influenza split-virion vaccine was approved in China in 2018. Given the limited sample size of its previous pivotal phase III clinical trial and the fact that all the included subjects were healthy, the safety and immunogenicity of products used in the elderly with chronic diseases or in poor health conditions lack pragmatic evidence. Especially for immunogenicity, the necessity of increasing the antigen dosage when used in older recipients to reach a protective immune response is still unclear. The emphasis of this study lies in demonstrating the safety and immunogenicity of the Hualan Bio quadrivalent influenza split-virion vaccine among the elderly population in pragmatic conditions to provide further evidence for influenza prevention and control in the elderly population.

## Subjects and methods

### Study design

This is an open-label, pragmatic post-marketing study. The objective of this study was to evaluate the safety and immunogenicity of a quadrivalent influenza vaccine, with the safety endpoints of the incidence of adverse events and serious adverse events, with immunogenicity endpoints as seroconversion rate, seroprotection rate, geometric mean titer (GMT) and geometric mean increase (GMI) of HI antibodies 30 days post-immunization.

This study was carried out in Shandong Province, China, and conducted by the Shandong Center for Disease Control and Prevention (CDC). Before initiation of the study, the protocol, informed consent form (ICF) and other information provided to recipients had been reviewed and approved by the Preventive Medical Ethical Committee of Shandong CDC (No. 2021-70).

### Study population

This study enrolled 1399 elderly subjects aged ≥ 60 years, without contraindications noted in the package insert of quadrivalent influenza vaccine. No rigorous physical or laboratory tests were conducted during the screening, because subjects in poor health condition were acceptable for this pragmatic study.

### Study vaccine

All screening-eligible subjects received one dose of quadrivalent influenza vaccine at the lateral deltoid muscle of the upper arm. The vaccine used in this study was a commercially available quadrivalent influenza split-virion vaccine produced by Hualan Bio that has been approved in China, that contains no adjuvant and 15 µg hemagglutinin per strain including A/H1N1, A/H3N2, B/Victoria and B/Yamagata. Prefilled syringes with 16 ± 1 mm length needles were used for vaccination, with 2/3 length of the needles injected in the lateral deltoid muscle of the upper arm. Batch No.: 202107B054. Stored and transported in 2 ~ 8℃ condition.

### Safety assessment

All vaccinated subjects were observed on-site for 30 min to assess immediate local and systemic adverse events, after which they were followed for 30 days for adverse event collection by recording on a contact card. Long-term safety observations were conducted within 31–180 days with a combination of methods of active monthly follow-up and self-reporting by subjects to collect serious adverse event (SAE) data.

Causality between adverse events and vaccination was analyzed in 5 degrees as: definitely-related, probably-related, possibly-related, likely-unrelated, and definitely-unrelated. Vaccination-related adverse events, including definitely-related, probably-related and possibly-related events, were referred to as adverse reactions. The severity of adverse events was categorized following the *Guidelines for the classification of adverse events in clinical trials of preventive vaccines* issued by the National Medical Products Administration (NMPA) in 2019 [21]. The collected adverse events were coded according to the Medical Dictionary for Regulatory Activities (MedDRA) and statistically analyzed for incidence and severity.

### Immunogenicity assessment

This study evaluated immunogenicity in a subgroup, endpoints of which included seroconversion rate and seroprotection rate of each type of HI antibody elicited by quadrivalent influenza vaccine in the elderly aged ≥ 60 years. Sample size of immunogenicity subgroup subjects was calculated with Confidence Interval of one Proportion method by using PASS 15.0. Assuming that the seroconversion rates of all types of HI antibodies exceeded 55%, the two-side confidence level 1-α = 0.95, the width of the confidence interval was 0.12, and the dropout rate was estimated as 20%, at least 347 subjects should undergo immunogenicity assessment. As a result, 350 subjects (25% of 1400), assigned randomly when enrolled, underwent venous blood sampling pre-vaccination and at 30 days after vaccination, to detect antibody titers. Immunogenicity evaluation was based on antibody titers against each subtype of influenza virus by micro-HI assay with serum separated from collected blood samples.

When statistically analyzed, referring to the NMPA *Technical Guidelines for Clinical Research of Seasonal Influenza Virus Vaccine* (Exposure Draft) [22], FDA *Clinical Data Needed to Support the Licensure of Seasonal Inactivated Influenza Vaccines* [17], and the EMEA *Note for Guidance on Harmonisation of Requirements for Influenza Vaccines* [16], seroprotection was defined as an HI antibody titer ≥ 1:40, and seroconversion was defined as an HI antibody titer change to ≥ 1:40 post-vaccination from baseline < 1:10 or a ≥ 4-fold increase in HI antibody titer post-vaccination from baseline ≥ 1:10. When the antibody titer was < 1:10, a titer of 1:5 was carried forward to calculate the GMT.

According to these guidance issued by NMPA, FDA and EMEA, quadrivalent influenza vaccines could be considered to have favorable immunogenicity among populations aged ≥ 60 years if at 30 days post-vaccination (1) The lower bound of the two-sided 95% confidence interval (CI) for the percentage of subjects achieving seroconversion for HI antibodies meet or exceed 30%; (2) The lower bound of the two-sided 95% CI for the percentage of subjects achieving an HI antibody titer ≥ 1:40 meet or exceed 60%; (3) The lower bound of the two-sided 95% CI for GMI > 2.0.

### Statistical analysis

We used SAS 9.4 for the statistical analysis of this study. The incidence of adverse events within 0–30 days post-vaccination, as the primary endpoint, and the incidence of SAEs within 0-180 days post-vaccination, as the secondary endpoint, were statistically described along with their Clopper-Pearson two-sided 95% CIs. For estimation of the primary immunogenicity endpoints, immunity assays and statistics were conducted among 350 subjects who were randomly assigned to an immune subset at the time of enrollment. The seroprotection rate and seroconversion rate were estimated, and the corresponding two-sided 95% CIs were derived from the Clopper-Pearson method. The GMTs and GMIs of the HI antibodies against each type of component (H1N1, H3N2, BV and BY) were calculated together with their two-sided 95% CIs.

The safety set (SS) includes data from all vaccinated subjects with at least one safety observation, while data from subjects with protocol violations were not excluded. The full analysis set (FAS) included data from all vaccinated subjects with detectable results from pre- or post-vaccination serum. The per-protocol set (PPS) includes data from subjects who underwent vaccination and blood sampling following predefined protocol requirements, with valid antibody detection results from pre-and post-vaccination serum.

### Serological methods

All the serum samples were treated by the National Institutes for Food and Drug Control in strict accordance with regulations and laboratory manuals. The micro-HI test was used to detect HI antibodies.

## Results

### Demographics and distribution

**Fig. 1:**
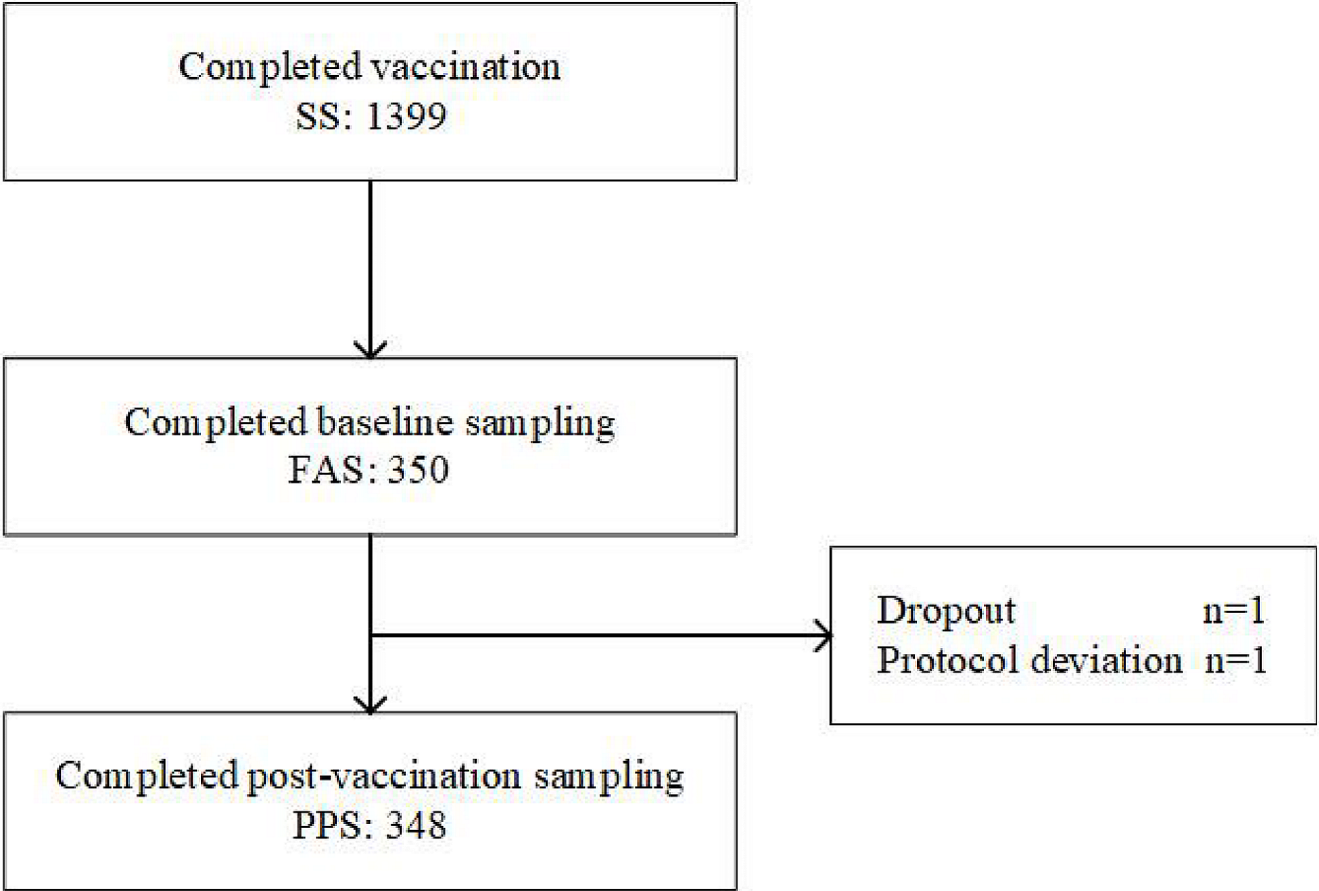
Analysis Set Distribution of the Subjects

A total of 1399 subjects, all Han Chinese, aged ≥ 60 years, were enrolled in this study and were vaccinated. All vaccinated subjects completed 30 min of on-site observation and were included in the SS analysis set. Among these, 350 subjects completed pre-vaccination blood sampling and were included in the FAS analysis set. After eliminating one subject who dropped out and one subject who reported protocol deviation, 348 subjects completed post-vaccination blood sampling and were included in the PPS analysis set (Fig. 1). The ages of all 1399 subjects ranged from 60 years to 96 years, with a median age of 69 years, and the sex distribution of all subjects was 666 males (47.61%) and 733 females (52.39%).

### Safety

Within 0–30 days post-vaccination, the incidences of overall adverse reactions, local adverse reactions and systemic adverse reactions were 3.79%, 2.86%, and 1.00%, respectively (Table 1).

Within 0–30 days post-vaccination, no grade 3 or worse adverse reactions developed. The incidences of Grade 2 and Grade 1 adverse reactions were 0.21% and 3.65%, respectively; the incidences of Grade 2 and Grade 1 local adverse reactions were 0.07% and 2.86%, respectively; and the incidences of Grade 2 and Grade 1 systemic adverse reactions were 0.14% and 0.86%, respectively (Table 2).

No vaccination-related serious adverse events developed during the whole study period, within 180 days post-vaccination.

By coding collected adverse reactions with MedDRA, all reactions could be categorized into 13 preferred terms (PTs). Symptoms developed in elderly subjects after being vaccinated with quadrivalent influenza vaccine included **common adverse reactions (1%~10%)**, such as vaccination site pain (2.5%); **uncommon adverse reactions (0.1%~1%)**, such as vaccination site pruritus (0.4%), cough (0.3%), vomiting (0.2%), vaccination site swelling (0.1%), vaccination site erythema (0.1%), headache (0.1%), fatigue (0.1%), and nausea (0.1%); **and rare adverse reactions (0.015 ~ 0.1%)**, such as pyrexia (0.07%), dizziness (0.07%), arthralgia (0.07%), and myalgia (0.07%) (Table 3).

**Table 1:** Incidence of adverse reactions within 0–30 days

Item	*N*	*n*	Incidences (95%CI)
**Overall ARs**	1399	53	3.79(2.85 ~ 4.93)
**Local ARs**	1399	40	2.86(2.05 ~ 3.87)
**Systemic ARs**	1399	14	1.00(0.55 ~ 1.67)

AR: adverse reaction; CI: confidence intervals;

N: subject number of analyzing set as denominator; n: number of subjects developed corresponding reactions

**Table 2:** Incidence of adverse reactions within 0–30 days by severity

Items	*N*	Grade 1		Grade 2		Grade 3	
		*n*	% (95%CI)	*n*	% (95%CI)	*n*	% (95%CI)
**Overall ARs**	1399	51	3.65(2.73 ~ 4.77)	3	0.21(0.04 ~ 0.63)	0	0.00(0.00 ~ 0.26)
**Local ARs**	1399	40	2.86(2.05 ~ 3.87)	1	0.07(0.00 ~ 0.40)	0	0.00(0.00 ~ 0.26)
**Systemic ARs**	1399	12	0.86(0.44 ~ 1.49)	2	0.14(0.02 ~ 0.52)	0	0.00(0.00 ~ 0.26)

AR: adverse reaction; CI: confidence intervals; N: subject number of analyzing set as denominator;

n: number of subjects developed corresponding reactions; %: incidence of subjects developed corresponding reactions

**Table 3:** Incidence of adverse reactions within 0–30 days by symptoms

Symptoms (PT)	*N*	*n*	Incidences (95%CI)
**Vaccination site pain**	1399	35	2.50(1.75 ~ 3.46)
**Vaccination site pruritus**	1399	6	0.43(0.16 ~ 0.93)
**Cough**	1399	4	0.29(0.08 ~ 0.73)
**Vomiting**	1399	3	0.21(0.04 ~ 0.63)
**Vaccination site swelling**	1399	2	0.14(0.02 ~ 0.52)
**Vaccination site erythema**	1399	2	0.14(0.02 ~ 0.52)
**Headache**	1399	2	0.14(0.02 ~ 0.52)
**Fatigue**	1399	2	0.14(0.02 ~ 0.52)
**Nausea**	1399	2	0.14(0.02 ~ 0.52)
**Pyrexia**	1399	1	0.07(0.00 ~ 0.40)
**Dizziness**	1399	1	0.07(0.00 ~ 0.40)
**Arthralgia**	1399	1	0.07(0.00 ~ 0.40)
**Myalgia**	1399	1	0.07(0.00 ~ 0.40)

PTs in descending order of incidence;

CI: confidence intervals;

N: subject number of analyzing set as denominator; n: number of subjects developed corresponding reactions

### Immunogenicity

**Fig. 2:**
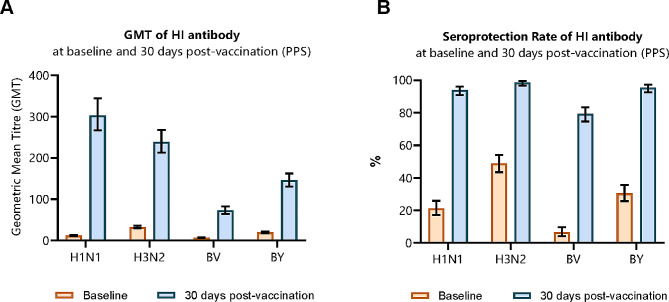
GMT and Seroprotection Rate of Hemagglutination Inhibition (HI) Antibodies. Legend: Data was analyzed among 348 subjects included in PPS. **A**: GMT of HI antibody at baseline and 30 days post-vaccination; **B**: Seroprotection Rate of HI antibody at baseline and 30 days post-vaccination. Seroprotection was defined as an antibody titer ≥ 1:40

**Fig. 3:**
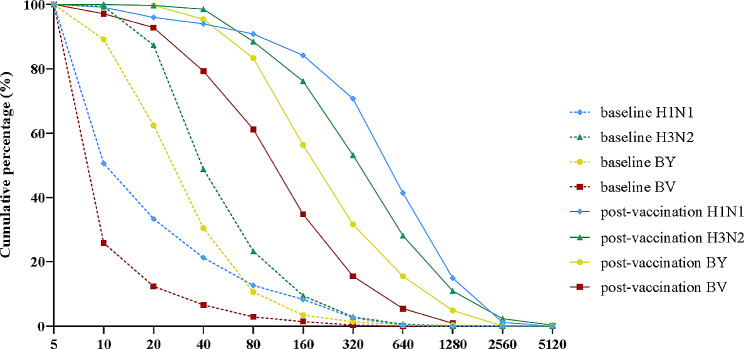
Reverse Cumulative Distribution Curve for Antibody Titer

At 30 days post-vaccination, the seroconversion rates (95% CI) of the HI antibody against the H1N1, H3N2, BV and BY subtypes were 87.64% (83.72%~90.91%), 75.57% (70.71%~80.00%), 73.28% (68.30%~77.85%), and 78.74% (74.06%~82.92%), respectively. The lower bounds of the two-sided 95% CIs for the seroconversion rate of each subtype exceeded 40%. The seroprotection rates (95% CIs) of the HI antibody against each subtype were 93.97% (90.92%~96.23%), 98.56% (96.68%~99.53%), 79.31% (74.67%~83.44%), and 95.40% (92.64%~97.35%), respectively (Fig. 2B). The lower bounds of the two-sided 95% CIs for the seroprotection rate of each subtype all exceeded 70% (Table 4). In most subjects, the HI antibody titers against H1N1 (70.69%) and H3N2 (53.16%) exceeded 1:320, those against BY (56.32%) exceeded 1:160, and those against BV (61.21%) exceeded 1:80 (Fig. 3).

At 30 days post-vaccination, the GMTs (95% CI) of the HI antibodies against H1N1, H3N2, BV and BY were 303.29 (267.03 ~ 344.37), 238.98 (212.93 ~ 267.76), 73.16 (64.86 ~ 82.49), and 145.74 (130.65 ~ 162.49), respectively (Fig. 2A), of which the GMIs (95% CI) were 24.80 (21.39 ~ 28.75), 7.26 (6.38 ~ 8.25), 10.39 (9.14 ~ 11.80), and 7.39 (6.57 ~ 8.31), respectively, of the baseline levels. The lower bounds of the two-sided 95% CIs for the GMI of each subtype all exceeded 2.5 (Table 5).

The results of the immunogenicity analysis of the FAS were in accordance with those of the PPS.

**Table 4:** Seroconversion and Seroprotection Rate on Day 30 post-vaccination (PPS)

Subtype	*N*	Seroconversion		Seroprotection	
		*n*	%	*n*	%
H1N1	348	305	87.64(83.72 ~ 90.91)	327	93.97(90.92 ~ 96.23)
H3N2	348	263	75.57(70.71 ~ 80.00)	343	98.56(96.68 ~ 99.53)
BV	348	255	73.28(68.30 ~ 77.85)	276	79.31(74.67 ~ 83.44)
BY	348	274	78.74(74.06 ~ 82.92)	332	95.40(92.64 ~ 97.35)

Analyzed with titer detected by hemagglutination inhibition assay

N: subject number of analyzing set as denominator;

n: number of subjects whose detection result of corresponding subtype meet seroconversion/seroprotection standard;

%: incidences of subjects whose detection result of corresponding subtype meet seroconversion/seroprotection standard

Seroprotection is defined as hemagglutination inhibition (HI) antibody titer ≥ 1:40

Seroconversion is defined as HI titer post-vaccination changed to ≥ 1:40 from baseline < 1:10 or ≥ 4-fold increase in HI titer post-vaccination from baseline ≥ 1:10

**Table 5:** GMT and GMI on Day 30 post-vaccination (PPS)

Subtype	*N*	GMT (95%CI)	GMI (95%CI)
H1N1	348	303.25(267.03 ~ 344.37)	24.80(21.39 ~ 28.75)
H3N2	348	238.78(212.93 ~ 267.76)	7.26(6.38 ~ 8.25)
BV	348	73.14(64.86 ~ 82.49)	10.39(9.14 ~ 11.80)
BY	348	145.70(130.65 ~ 162.49)	7.39(6.57 ~ 8.31)

Analyzed with titer detected by hemagglutination inhibition assay

N: subject number of analyzing set as denominator; CI: confidence intervals;

GMT: Geometric mean titer; GMI: Geometric mean increase fold at 30 days post-vaccination compared to baseline level

## Discussion

This open-label phase IV study was carried out from 2021 to 2023, in 4900 subjects aged ≥ 3 years population, including 1399 older adults. We published results of the elderly group separately because the health status of this age group in practical condition differs from that in strict randomized controlled trials (RCTs) to larger extent compared to other age groups, and the elderly are prone to face more risk when suffering from influenza. This study aimed to provide pragmatic post-marketing evidence of quadrivalent influenza vaccine used in older recipients for health system policy makers to guide the delivery of influenza vaccines in the elderly. By loosening the eligibility criteria, compared to that of previous RCTs, this study enrolled subjects aged ≥ 60 years without contraindications noted in the package insert of quadrivalent influenza vaccines. Elderly individuals with chronic disease or in poor health were accepted to enrolment. This licensed vaccine exhibited a favorable safety profile after inoculation in the target population. Adverse reactions that developed within 30 days post-vaccination were mostly limited to Grade 1, and no Grade 3 or worse adverse reactions developed. The frequency of adverse events was relatively lower than that of the other study conducted in population aged 3–60 years, because elderly people are less sensitive to discomfort [23]. Compared to clinical trials conducted in China with the other local-unlicensed product, the relatively low incidences of local reactions and systemic reactions could be attributed to the psychological presupposition of subjects for licensed product [24]. Blood samples from 348 subjects were collected at 30 days post-vaccination for HI assays to evaluate immunogenicity. The lower bounds of the two-sided 95% CIs for seroconversion rates, seroprotection rates, GMTs and GMIs of each subtype all exceed standards issued by the NMPA, FDA and EMEA.

Going through the 3-year epidemic of the novel coronavirus, the societal impact and disease burden of respiratory infectious disease on the elderly population have been fully recognized, and people’s attention and awareness of vaccination have greatly improved. It was pointed out in China’s 7th population census that, by the end of 2020, the elderly population aged 60 and above had reached 264 million, accounting for 18.7% of the entire population, which will keep increasing [25]. The aging of the Chinese population has become a serious social and public health issue concerning the elderly and merits close attention.

It is generally believed in developed countries that the immunogenicity elicited by currently licensed influenza split-virion vaccines (15 µg/subtype/0.5 ml/dose) when used in older populations is relatively weak to generate ideal immune protection; thus, influenza vaccines specifically for the elderly use were developed by increasing the dosage or adding adjuvants [5]. However, this study demonstrated that the Hualan Bio quadrivalent influenza vaccine manifests favorable immunogenicity and safety profiles not only in pivotal phase III trial, but also among elderly individuals aged ≥ 60 years in pragmatic conditions, the immunogenicity of which exceeds standards issued by the FDA and EMEA. In addition to product characteristics, influenza epidemiology in China might also be involved. The possibility that older Chinese people have stronger immune memory against influenza virus than people in developed countries cannot be ruled out.

Currently, influenza vaccines of different production platforms have been licensed worldwide, including inactivated vaccine, subunit vaccine, live-attenuated vaccine, etc. Since the inactivated split vaccine was first developed in the 1960s, it has accumulated a lot of production and clinical use experience, and sufficient comprehensive historical data on vaccine safety, immunogenicity and protective efficacy [1]. Compared to other platform, inactivated vaccine has advantages in product stability, and mature production process and quality control standard. Compared with subunit vaccines, the antigen of inactivated split vaccines has a more complete spatial domain, which is expected to perform better in inducing immune response. Live attenuated vaccines are mainly administered by nasal spray, which imitates natural infection stimulating both humoral and mucosal immunity. However, recipients face risk of using live viruses for immunization, such as virulent enhancement and viral shedding. Production of inactivated split vaccine relies on chicken embryo cells (CEC), supply of which is sufficient for influenza prevention and control of average pandemic intensity. However, main surface antigen of influenza virus is highly variable. In circumstance that a new type of viruses dominantly circulating in summer caused by antigenic shift or antigenic drift, high-temperature will increase the risk of pathogen contamination in CEC supply, which can affect production of the inactivated split vaccine. The mRNA platform will not be limited by the CEC supply and perform potential for development in this particular circumstance [26]. However, there is no strong confirmatory clinical trial results of influenza mRNA vaccine currently, and its long-term safety needs to be further verified in the future.

Further efficacy study of this quadrivalent influenza split-virion vaccine is under planning to provide evidence for establishing the correlation between vaccine effectiveness and protective immune response level, so as to provide a more well-rounded scientific basis for influenza prevention and control strategy. In addition to the actual application population factors concerned in this study, as the proportion of vaccinated individuals in the population increases, the spread of pathogens in the population will be limited, which will bring additional indirect benefits to unvaccinated individuals, namely herd immunity [27]. This may constitute the objective of future studies, that network model and cluster randomization study are potentially valuable in herd effect assessment to quantify the extent to which this indirect protection influences the influenza epidemic [28, 29].

## Conclusion

This study strongly demonstrated that the Hualan Bio quadrivalent influenza vaccine raises no safety concerns and could elicit a protective titer of HI antibodies against vaccine-matched subtypes at 30 days post-vaccination in older adults. The vaccine-acquired immunogenicity profile meets the standards issued by the NMPA, FDA and EMEA, even without increasing the dosage for the elderly specifically. Taken together, the immunogenicity and safety results of this study suggest that the Hualan Bio quadrivalent influenza split-virion vaccine has the potential to further address the disease burden of influenza, especially in elderly people. In addition, it will be worthwhile to conduct additional studies to evaluate herd protection to more fully understand the performance of the vaccine under real-world conditions.

## Data Availability

No datasets were generated or analysed during the current study.

## Abbreviations

CBER: Center for Biologics Evaluation and Research
CDC: Center for Disease Control and Prevention
CEC: chicken embryo cells
CI: confidence interval
EMEA: the European Medicines Evaluation Agency
FAS: full analysis set
FDA: Food and Drug Administration
GMI: geometric mean increase
GMT: geometric mean titer
HI: hemagglutination inhibition
ICF: informed consent form
MedDRA: medical dictionary for regulatory activities
NMPA: National Medical Products Administration
PPS: per protocol set
PT: preferred term
RCT: randomized controlled trial
SAE: serious adverse event
SS: safety set

## References

[1] Kim YH, Hong KJ, Kim H, Nam JH. Influenza vaccines: past, present, and future. Rev Med Virol. 2021. 10.1002/rmv.2243. 33949021 10.1002/rmv.2243 PMC8209895

[2] Paules C, Subbarao K. Influenza Lancet. 2017. 10.1016/s0140-6736(17)30129-0. 28302313 10.1016/s0140-6736(17)30129-0

[3] https://www.who.int/news-room/fact-sheets/detail/influenza-(seasonal). Accessed.

[4] Somes MP, Turner RM, Dwyer LJ, Newall AT. Estimating the annual attack rate of seasonal influenza among unvaccinated individuals: a systematic review and meta-analysis. Vaccine. 2018. 10.1016/j.vaccine.2018.04.063. 29716771 10.1016/j.vaccine.2018.04.063

[5] Quach HQ, Kennedy RB. Enhancing immunogenicity of Influenza Vaccine in the Elderly through Intradermal Vaccination: A literature analysis. Viruses; 2022. 10.3390/v14112438. 10.3390/v14112438 PMC9698533 36366536

[6] Yu H, Huang J, Huai Y, Guan X, Klena J, Liu S, et al. The substantial hospitalization burden of influenza in central China: surveillance for severe, acute respiratory infection, and influenza viruses, 2010–2012. Influenza and Other Respiratory Viruses; 2014. 10.1111/irv.12205. 10.1111/irv.12205 PMC4177798 24209711

[7] Iuliano AD, Roguski KM, Chang HH, Muscatello DJ, Palekar R, Tempia S, et al. Estimates of global seasonal influenza-associated respiratory mortality: a modelling study. Lancet. 2018. 10.1016/s0140-6736(17)33293-2. 29248255 10.1016/s0140-6736(17)33293-2 PMC5935243

[8] Li L, Liu Y, Wu P, Peng Z, Wang X, Chen T, et al. Influenza-associated excess respiratory mortality in China, 2010–15: a population-based study. Lancet Public Health. 2019. 10.1016/s2468-2667(19)30163-x. 31493844 10.1016/s2468-2667(19)30163-x PMC8736690

[9] Wang H, Fu C, Li K, Lu J, Chen Y, Lu E, et al. Influenza associated mortality in Southern China, 2010–2012. Vaccine. 2014. 10.1016/j.vaccine.2013.12.013. 24370709 10.1016/j.vaccine.2013.12.013

[10] Feng L, Shay DK, Jiang Y, Zhou H, Chen X, Zheng Y, et al. Influenza-associated mortality in temperate and subtropical Chinese cities, 2003–2008. Bull World Health Organ. 2012. 10.2471/blt.11.096958. 22511824 10.2471/blt.11.096958 PMC3324869

[11] Wu P, Goldstein E, Ho LM, Yang L, Nishiura H, Wu JT, et al. Excess mortality Associated with Influenza A and B Virus in Hong Kong, 1998–2009. J Infect Dis. 2012. 10.1093/infdis/jis628. 23045622 10.1093/infdis/jis628 PMC3502382

[12] Yang L, Ma S, Chen PY, He JF, Chan KP, Chow A, et al. Influenza associated mortality in the subtropics and tropics: results from three Asian cities. Vaccine. 2011. 10.1016/j.vaccine.2011.09.071. 21959328 10.1016/j.vaccine.2011.09.071 PMC7115499

[13] Demicheli V, Jefferson T, Di Pietrantonj C, Ferroni E, Thorning S, Thomas RE, et al. Vaccines for preventing influenza in the elderly. Cochrane Database Syst Reviews. 2018. 10.1002/14651858.CD004876.pub4. 10.1002/14651858.CD004876.pub4 PMC6491101 29388197

[14] Beyer WEP, McElhaney J, Smith DJ, Monto AS, Nguyen-Van-Tam JS, Osterhaus ADME. Cochrane re-arranged: support for policies to vaccinate elderly people against influenza. Vaccine. 2013. 10.1016/j.vaccine.2013.09.063. 24095882 10.1016/j.vaccine.2013.09.063

[15] Che X, Liu Y, Gu W, Wang F, Wang J, Jiang W, et al. Analysis on the intention and influencing factors of free influenza vaccination among the elderly people aged 70 and above in Hangzhou in 2022. Front Public Health. 2023. 10.3389/fpubh.2022.1052500. 36684888 10.3389/fpubh.2022.1052500 PMC9853049

[16] EMEA, NOTE FOR GUIDANCE ON HARMONISATION OF REQUIREMENTS FOR INFLUENZA, VACCINES. 1997. https://www.ema.europa.eu/en/harmonisation-requirements-influenza-vaccines. Accessed 04 Mar 2024.

[17] FDA-CBER. Clinical Data Needed to Support the Licensure of Seasonal Inactivated Influenza Vaccines. 2007. https://www.fda.gov/regulatory-information/search-fda-guidance-documents/clinical-data-needed-support-licensure-seasonal-inactivated-influenza-vaccines. Accessed 04 Mar 2024.

[18] ECDC, Systematic review of the efficacy, effectiveness and safety of newer and enhanced seasonal influenza vaccines for the prevention of laboratory-confirmed influenza in individuals aged 18 years and over. 2020. 10.2900/751620.

[19] Stassijns J, Bollaerts K, Baay M, Verstraeten T. A systematic review and meta-analysis on the safety of newly adjuvanted vaccines among children. Vaccine. 2016. 10.1016/j.vaccine.2015.12.024. 26740250 10.1016/j.vaccine.2015.12.024

[20] Grohskopf LA, Blanton LH, Ferdinands JM, Chung JR, Broder KR, Talbot HK, et al. Prevention and Control of Seasonal Influenza with vaccines: recommendations of the Advisory Committee on Immunization practices — United States, 2022–23 influenza season. MMWR Recomm Rep; 2022. 10.15585/mmwr.rr7101a1. 10.15585/mmwr.rr7101a1 PMC9429824 36006864

[21] NMPA. Guidelines for the classification of adverse events in clinical trials of preventive vaccines. 2019. https://www.nmpa.gov.cn/xxgk/ggtg/ypggtg/ypqtggtg/20191231111901460.html. Accessed 04 Mar 2024.

[22] NMPA. Technical Guidelines for Clinical Research of Seasonal Influenza Virus Vaccine (Exposure Draft). 2021. https://www.cde.org.cn/main/news/viewInfoCommon/237d5f7de6bcfcd08037dcce873794f3. Accessed 04 Mar 2024.

[23] Huang X, Fan T, Li L, Nian X, Zhang J, Gao X, et al. Safety and immunogenicity of a quadrivalent, inactivated, split-virion influenza vaccine (IIV4-W) in healthy people aged 3–60 years: a phase III randomized clinical noninferiority trial. Human Vaccines & Immunotherapeutics; 2022. 10.1080/21645515.2022.2079924. 10.1080/21645515.2022.2079924 PMC9621009 35714276

[24] Liu X, Park J, Xia S, Liang B, Yang S, Wang Y, et al. Immunological non-inferiority and safety of a quadrivalent inactivated influenza vaccine versus two trivalent inactivated influenza vaccines in China: results from two studies. Human Vaccines & Immunotherapeutics; 2022. 10.1080/21645515.2022.2132798. 10.1080/21645515.2022.2132798 PMC9746500 36328438

[25] National Bureau of Statistics. State Statistics Bureau Bulletin of the seventh national population Census (No.5). 2021. https://www.stats.gov.cn/sj/tjgb/rkpcgb/qgrkpcgb/202302/t20230206_1902005.html. Accessed 04 Mar 2024.

[26] Kackos CM, DeBeauchamp J, Davitt CJH, Lonzaric J, Sealy RE, Hurwitz JL, et al. Seasonal quadrivalent mRNA vaccine prevents and mitigates influenza infection. npj Vaccines. 2023. 10.1038/s41541-023-00752-5. 37828126 10.1038/s41541-023-00752-5 PMC10570305

[27] Mooring EQ, Bansal S. Increasing herd immunity with influenza revaccination. Epidemiol Infect. 2015. 10.1017/s0950268815002253. 26482721 10.1017/s0950268815002253

[28] Hitchings MDT, Lipsitch M, Wang R, Bellan SE. Competing effects of Indirect Protection and Clustering on the power of cluster-randomized controlled vaccine trials. Am J Epidemiol. 2018. 10.1093/aje/kwy047. 29522080 10.1093/aje/kwy047 PMC6070038

[29] Law KB, Peariasamy KM, Mohd Ibrahim H, Abdullah NH. Modelling infectious diseases with herd immunity in a randomly mixed population. Sci Rep. 2021. 10.1038/s41598-021-00013-2. 34663839 10.1038/s41598-021-00013-2 PMC8523531

